# Influence of Ion Strength and pH on Thermal Stability of Yeast Formate Dehydrogenase

**Published:** 2010-07

**Authors:** V.I. Tishkov, S.V. Uglanova, V.V. Fedorchuk, S.S. Savin

**Affiliations:** Department of Chemical Enzymology, Faculty of Chemistry, Lomonosov Moscow State University; Innovations and High Technologies MSU Ltd.; Bach Institute of Biochemistry, Russian Academy of Sciences; Emanuel Institute of Biochemical Physics, Russian Academy of Sciences

**Keywords:** formate dehydrogenase, Candida boidinii, thermal inactivation, ionic strength, stabilization

## Abstract

The kinetics of the thermal inactivation of recombinant wild–type formate dehydrogenase from * Candida boidinii * yeast was studied in the temperature range of 53–61^o^C and pH 6.0, 7.0, and 8.0. It was shown that the loss of the enzyme’s activity proceeds via a monomolecular mechanism. Activation parameters ∆Н^­^ and ∆S^­^ were calculated based on the temperature relations dependence of inactivation rate constants according to the transition state theory. Both parameters are in a range that corresponds to globular protein denaturation processes. Optimal conditions for the stability of the enzyme were high concentrations of the phosphate buffer or of the enzyme substrate sodium formate at pH = 7.0.

## INTRODUCTION

NAD^+^–dependent formate dehydrogenase (EC 1.2.1.2, FDH) belongs to the superfamily of D–specific dehydrogenases of 2–hydroxyacids [1]. Because of the simplicity of the catalyzed reaction, which is a simple transfer of the hydride ion in the active site between the formate and the C4 atom of the nicotinamide ring with no acidic–basic catalysis involved, FDH is used as a model system for studying the enzyme catalytic mechanism of the whole superfamily.

The most thoroughly studied FDHs are the ones derived from bacterium *Pseudomonas * sp.101 (PseFDH) and from yeast * Candida boidinii *; (CboFDH). Studies of both of these enzymes started almost simultaneously in the early 1970s. There is now a large number of mutant forms of these enzymes [2], and their quaternary structure has been determined using X–ray analysis [3–5]. Plant FDHs, which are localized in the mitochondria, are also an interesting topic for research. * Escherichia coli * strains which produce recombinant plant FHDs from * Arabidopsis thaliana * and soy * Glycine max * have been constructed in our laboratory [6]. Crystals of these FHDs were obtained very recently, and the structures of the enzymes have been mapped [7].

One of the most important characteristics of an enzyme is its thermal stability. This is a very important parameter for the practical use of an enzyme. We have conducted systematic studies of the thermal inactivation of the wild–type * Pseudomonas * sp.101 FDH, as well as that of mutant forms of this enzyme at various temperatures, pH, and concentrations of a phosphate buffer [2, 8]. We were able to show that an increase in the solution’s ionic strength causes the observed inactivation rate constant to reach its maximum and that an elevated (several fold) stability of the enzyme can be observed at high concentrations of the phosphate buffer [8]. There are no such data for yeast * C. boidinii *; FDH in the available literature; however, indirect data indicate that the effect of the surrounding solution on the stability of CboFDH may be even stronger. For instance, the half–time inactivation period of the enzyme is a few days during storage at +4^0^С in a 0.1 М phosphate buffer, рН7.0; that is why CboFDH samples should be stored in 40% glycerol at –20^0^С. However, the enzyme can remain activitve for several weeks during the synthesis of * L* –tert–leucine at a temperature of 25–30^0 ^С in a flow membrane reactor [9]. This process involves a high concentration of the CboFDH substrate ammonium formate, which suggests that the presence of the substrate stabilizes the enzyme.

The aim of this work was to carry out a systematic study of the stability of recombinant wild–type CboFDH at increased temperatures and рН 6.0–7.0, as well as at varying concentrations of phosphate buffer and the substrate of the enzyme, sodium formate.

## EXPERIMENTS

For this work we used a preparation of recombinant formate dehydrogenase originating from wild–type * Candida boidinii yeast. * Cultivation of * E. coli* (BL21(DE3)/pCboFDH) cells expressing the * C. boidinii FDH* was performed at 25°C in 250 ml or 1l shaker flasks with baffles using 50 or 250 ml of medium, respectively. The medium consisted of 16 g/l tryptone, 1 0 g/ l of yeast extract, 1 g/l of sodium chloride, 1.5 g/l of H2NaPO4, 1 g/l of HK2PO4, 100 micrograms/ml of ampicillin, and 25 micrograms/ml of chloramphenicol. The volume of the bacterial inoculate was equal to 10–15% of the medium volume. Lactose was used for the unduction of FDH biosynthesis and an inducer was added to a final concentration of 20 mg/ml when absorbance of the cell suspension at 600 nm ( * А *_ 600 _ ) reached the value 0.5 – 0. 7 . The cells were then grown in maximum aeration overnight. Then, the cells were spun down on a Beckman J–21 (United States) centrifuge at 8000 rpm for 20 minutes at 4°С. Recombinant CboFDH was then purified according to the standard protocol developed for * Pseudomonas * sp.101 FDH [10]. The enzyme purification procedure involved the destruction of the 10% w/v cell suspension in a 0.1 М potassium–phosphate buffer, 0.02 M EDTA, and рН 8.0 using a Braun Sonic ultrasound disintegrator (Germany) at 0°С, the precipitation of some of the ballast proteins with ammonium sulfate (35% of saturation), hydrophobic chromatography on a Fast Protein Liquid Chromatography (FPLC) apparatus (Pharmacia Biotech, Sweden) using a column with Phenyl Sepharose Fast Flow from the same company, and gel filtration on a Sephacryl S200 column. The obtained preparations were at least 95% pure as assayed by an analytical gel electrophoresis in a 12% polyacrylamide gel in denturating conditions.

**Formate dehydrogenase activity assay **. FDH activity was measured spectrophotometrically by monitoring the accumulation of NADH at a wavelength of 340 nm ( ε _340_ = 6 220 М^–1 ^cm^–1^ ) on a Schimadzu UV 1601PC spectrophotometer at 30°C in a 0.1 М sodium–phosphate buffer, рН 7.0. Saturated NAD^+^ and formate concentrations in the cuvette were 1.5 мМ and 0.3 М, respectively.

**Study of the thermal inactivation of recombinant CboFDH**. The thermal stability of the enzyme was assayed in a sodium phosphate buffer at a given concentration in the 0.01–1.5 М (pН 6.0–8.0) range and supplemented by 0.01 М EDTA and, depending on the type of experiment, 0.1–2.5 М of sodium formate. Each experiment used a series of 1.5 ml plastic tubes with 100 microliters of the enzyme solution (0.2–0.25 mg/ml). The tubes were placed into a preheated water thermostat (53–61°C, accuracy ±0.1°C). The tubes were taken out at regular intervals, transferred into an ice bath for 5 min, and then spun down for 2 min at 12 000 rpm in an Eppendorf 5415D centrifuge. The remaining FDH activity was assayed as described above. The thermal inactivation rate constant *k_in_* was determined as the slope of the plot of a natural logarithm of the remaining activity value versus time (semilogarithmic coordinates ln( * А/A* _0 _ ) – * t * ) using the linear regression method available in the Origin 7.0 software package.

## RESULTS AND DISCUSSION

**Effect of pH on the Thermal Inactivation Rate of Recombinant** *C. boidinii FDH*.

Previous studies of bacterial FDH from * Pseudomonas * sp *. * 101 at temperatures above 37°С [2, 8] have demonstrated the following:

1. The thermal inactivation of the enzyme is irreversible;
2. The time-course of loss of enzymatic activity fits the kinetics of a first order reaction;
3. The observed first order inactivation rate constant does not depend on the concentration of the enzyme,which means that the inactivation of bacterial FDH at high temperatures is, in fact, a true monomolecular process.

**Fig. 1:**
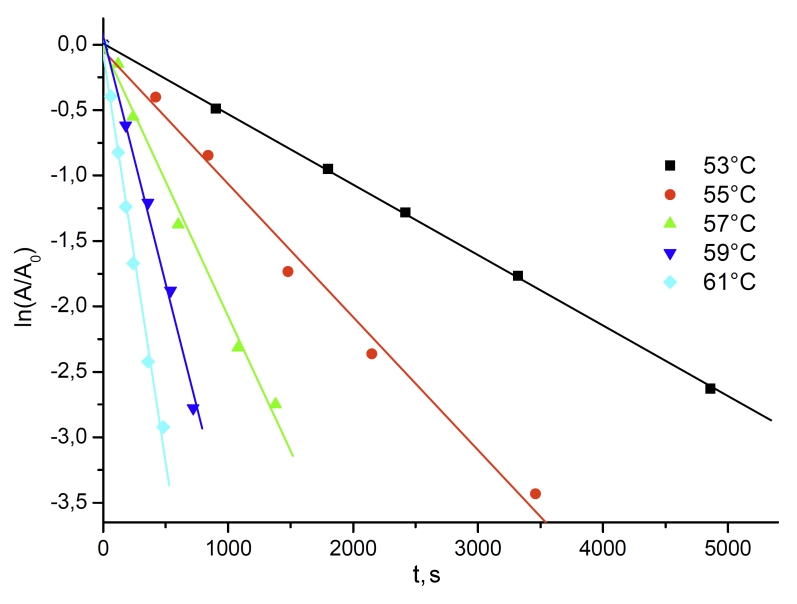
Dependence of CboFDH residual activity on time as plot ln(А/А_0_) – t at different temperatures. 0.1M sodium phosphate buffer, pH 6.0.

The thermal inactivation of recombinant CboFDH was studied at a temperature interval of 53–61°С and pH values of 6.0, 7.0, and 8.0. Fig. 1 shows the dependence of the remaining enzymatic activity on time at various temperatures in a 0.1 М phosphate buffer, рН 6.0.

As can be seen in Fig. 1, the relations are linear in semilogarithmic coordinates (ln( * A/A *_ 0 _ ) – * t* ). The slope of the lines (which is the inactivation rate constant * k* _in _ ) does not depend on the concentration of the enzyme in a range of 0.08–1.5–mg/m l. The linear character of the relation between the remaining enzymatic activity and time in semilogarithmic coordinates and the constant value of the observed inactivation rate constant at various enzyme concentrations indicate that the thermal inactivation of CboFDH, like that of bacterial enzymes, is a monomolecular process, which means that it is a single–stage process with no preceding dissociation of the dimeric enzyme into its separate subunits.

A study of the loss of activity of CboFDH at рНs 7.0 and 8.0 showed that the thermal inactivation of the enzyme is also a monomolecular process. Table 1 shows the values of the observed first–order inactivation rate constants for * C. boidinii yeast FDH* in a 0.1 М sodium–phosphate buffer at a varying pH.

Bacterial FDH are notably more stable than CboFDH. For instance, at рН 7.0 and 61°С, the inactivation rate constant for CboFDH was 2.26 × 10^–3^ sec^–1^, while the appropriate constant for wild–type PseFDH at this temperature was 1. 3 × 10^–4^ sec^–^^1^ [2], which is almost 20–fold less.

**Table 1:** Table 1. Observed inactivation rate constants for recombinant formate dehydrogenase from c.boidinii in a 0.1M sodium phosphate buffer at different pHs.

pH	k_in_10^6^, s^-1^
53°С	55°С	57°С	59°С	61°С
6.0	539 ± 4	1020 ± 50	2070 ± 80	3800 ± 200	6200 ± 200
7.0	28 ± 1	97 ± 3	370 ± 20	1050 ± 50	2260 ± 90
8.0	290 ± 9	1000 ± 10	2570 ± 40	6350 ± 200	12000 ± 400

**Table 2:** Table 2. Activation parameters for the thermal inactivation of CboFDH at different pHs.

pH	∆H^≠^ , kJ·mole^-1^	∆S^≠^ , J·K-1 mole^-1^
6.0	280 ± 9	500 ± 30	7.0	500 ± 30	1360 ± 90	8.0	420 ± 30	1240 ± 80

**Activation Parameters of the CboFDH Thermal Inactivation Process **. The fact that the yeast FDH is inactivated via a monomolecular process at the studied temperatures and pH range makes it possible to use the transition state theory to analyze this process.

According to the trasition state theory, the equation that describes the relation between the observed first–order rate constant and the temperature can be represented thus:

**Formula 1:** 

where * k_B_* and * h * are the Boltzmann and Plank constants, respectively; * R * is the universal gas constant, and * Т * is the temperature in Kalvin degrees; and *∆* *G^­^* , * Δ* *H* ^­, ^ and * ∆* *S^­^* are the changes of activation energy, enthalpy, and entropy, respectively.

Equation (1) can be transformed into the following linear form:

**Formula 2:** 

**Fig. 2:**
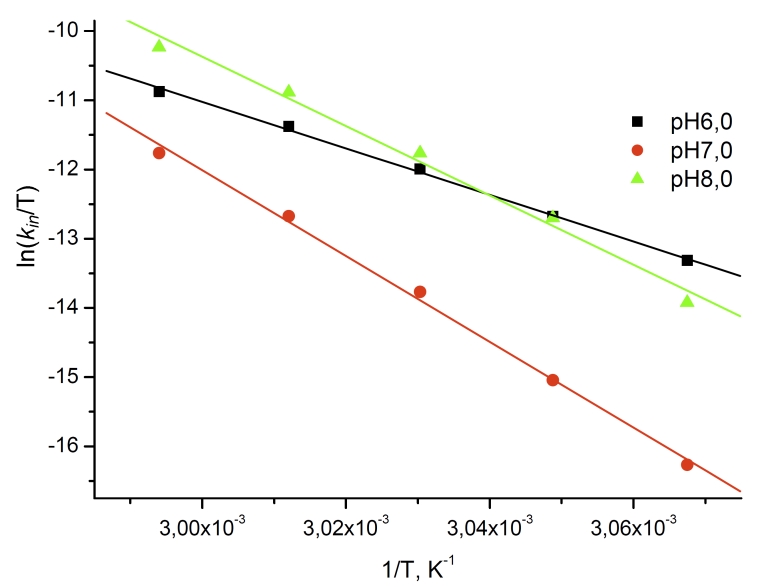
Temperature dependence of the first order inactivation rate constant for recombinant wild-type CboFDH at different pH values in coordinates ln(k_in_/T) – 1/T. 0.1М sodium phosphate buffer.

As can be seen in Fig. 2, the experimentally determined dependences between the observed inactivation rate constants and temperature in ln( * k_ин_* / * T * ) – 1/ * T * coordinates fit into straight lines, which means that these relations can be described by the trasition state–theory equation. Values of *Δ* *H ^ ­ ^* .were determined from the slope of the lines in Fig. 2.

The value of *∆S^≠^* can be calculated by approximating the line in Fig. 2 onto the zero point of the ordinate axis. However, this procedure will result in serious errors, since one has to do an approximation to a very large distance, the value ln( * k_B_* / * h * ) must also be subtracted from the value of intercept. The *∆* *S^­^* can be obtained much more accurately from the slope of the plot relating * ∆ G^­^* and *Т * according to the following equation:

*∆G^≠^*=*∆H^≠^*-T*∆S^≠^*.

A calculation of the activation free energy involves the following expression:

**Formula 3:** 

The values of * ∆* *H^­ ^* and *∆* *S^­^* for three ** pH values are presented in Table 2.

In most cases the inactivation of the enzyme at high temperatures is caused by the denaturation of the protein globule. Thermal denaturation is a cooperative process, and it must be accompanied by increases in both the * ∆* *Н^­^* and *∆* *S^­ ^* values. These increases are much larger (tenfold or more) than the similar ones seen in chemical reactions, which can be seen for the obtained * ∆* *Н^­^* and * ∆* *S^­^* values for the thermal inactivation of CboFDH (Table 2). Notably, similar values of activation parameters were obtained during a study of thermal inactivation for various bacterial formate dehydrogenases [8].

As is clear from these data, pH has a significant effect on the thermal inactivation of * C. boidinii *; FDH. The enzyme is most stable at рН 7.0. An increase or decrease in the pH value causes the rapid destabilization of the protein globule, which in turn causes a 3–20–fold increase in * k_in_* , depending on the temperature (Table 1). Notably, the relation between the temperature and *k_in_* upon a varying pH is somewhat different. The relation changes quickly at pH 7.0 and is slower at pH 6.0 (Fig. 2). This seems to be due to the altered ionization of charged groups in the protein globule (such as the loss of a positive charge by a histidine residue at рН ≥7.0 or the appearance of a positive charge on a histidine residue at рН ≤7.0), which leads to a decrease in the number of oppositely charged groups taking part in electrostatic interactions or creates repulsive interactions between residues with the same charge. For instance, according to an X–ray analysis, the ND1 atom of the His57 residue of CboFDH is located at a distance of 2.82 Å from the NZ atom of the amino group of the Lys2 residue of the same subunit, and the distance between the ND1–atom of the His126 residue of one subunit and the NE–atom of the guanidine group of the Arg136 of another subunit is 3.61 Å (structure 2FSS.PDB). The apo form of a CboFDH molecule might contain four electrostatic bonds between His residues and the carboxy groups of Asp and Glu residues. The distance between the interacting entities is less than 4 Å . Moreover, it is important to bear in mind that alterations in even a single group’s ionization can cause considerable conformational changes in a protein globule.

**The Dependence of CboFDH Thermal Stability on Concentration of Phosphate Ions**

As was mentioned above, electrostatic interactions play a very important role in the formation of native protein conformation. The effectiveness of these interactions is weakened in solutions with a high ionic strength. In order to analyze the effect that electrostatic interactions have on the stability of * C. boidinii yeast FDH, * we analyzed the thermal inactivation of the enzyme in solutions with varying concentrations of phosphate. We chose the phosphate ion because of its large size, which prevents it from penetrating the protein globule. Therefore, it should only disrupt the ionic interactions on the protein surface and thus have no effect on the structure of the protein globule itself. For comparison, we performed the same analysis with mutant FDH from * Pseudomonas* sp * . * 101 GAV (PseFDH) at pH 8.0.

**Fig. 3:**
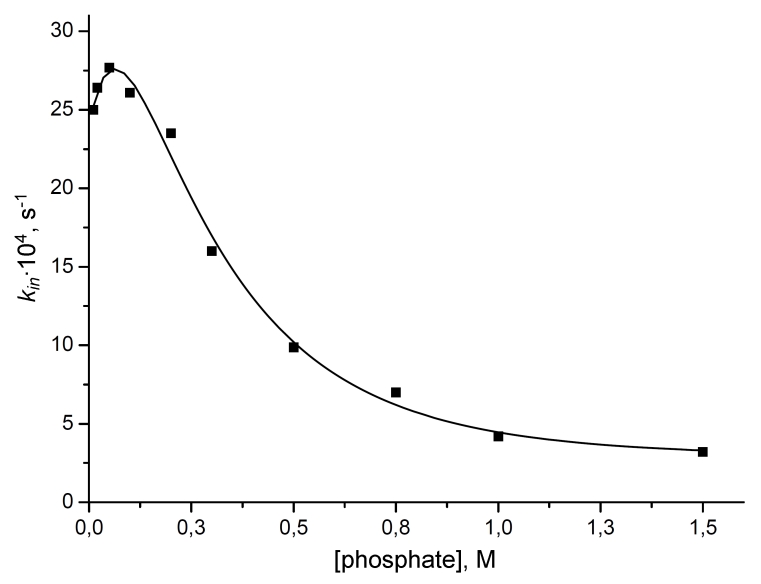
Influence of sodium phosphate buffer concentration on the inactivation rate constant of CboFDH. 0.01 – 1.5M sodium phosphate buffer, pH 7.0, 61°C.

Fig. 3 shows the relationship between the inactivation rate constant of the * C. boidinii *; FDH and the concentration of the phosphate buffer at рН 7.0. At first, increasing the ionic strength of the solution lowers the stability of the enzyme, which can be attributed to an increase in the dielectric permittivity of the solution and the disruption of electrostatic interactions. However, a further increase in the salt concentration causes the enzyme stability to increase (approximately seven– fold in the overall stabilization). A similar relation is seen at рН 8.0; however, the stabilization effect is much more pronounced, almost 100–fold.

The effect of high concentrations of the phosphate buffer on the thermal stability of recombinant wild–type PseFDH has been studied before [8]. For this enzyme, the maximum value of the inactivation rate constant was observed at a higher concentration of the phosphate buffer (0.2 М), the destabilization effect was stronger (two–fold) compared to the CboFDH (about 11%), and high concentrations of the phosphate buffer (>1 М) did not have any stabilizing effect when compared to lower concentrations (0.05 М).

**Fig. 4:**
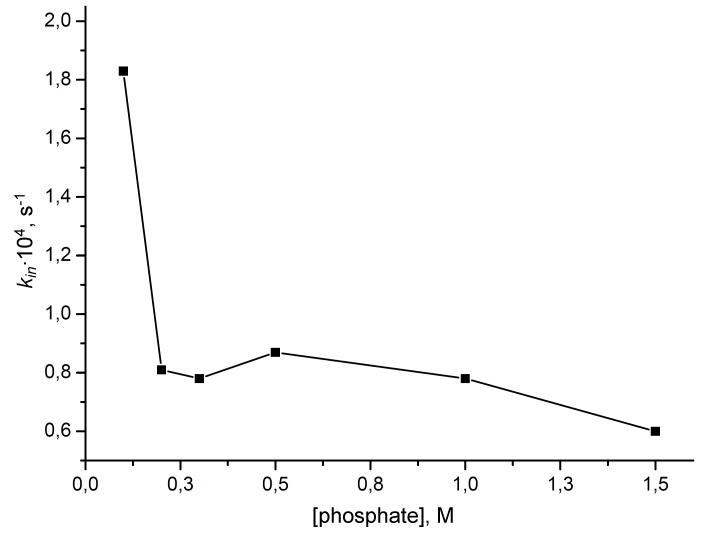
Influence of the sodium phosphate buffer concentration on the inactivation rate constant of PseFDH GAV. 0.1 – 1.5M sodium phosphate buffer, pH 8.0, 61°C.

Fig. 4 shows the relationship between the inactivation rate constant and the concentration of the phosphate ion for the mutant FDH from *Pseudomonas * sp * . * 101, GAV version (PseFDH GAV) at pH 8.0 and 61°С. The increase in the salt concentration causes only weak stabilization in the case of PseFDH GAV: only 3–fold, as compared to 100–fold for CboFDH at рН 8.0. However, PseFDH GAV is much more stable as it is; even a 100–fold increase in CboFDH stability is not enough to render it as stable as the bacterial enzyme.

**Fig. 5:**
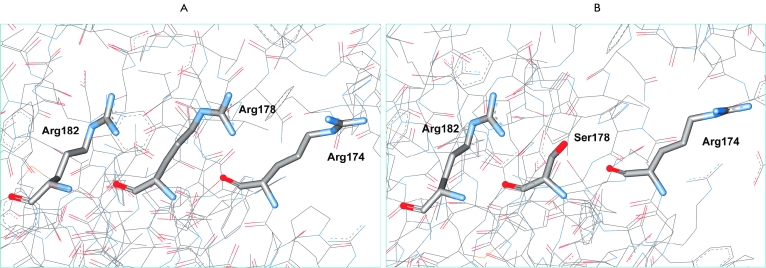
(A) Spatial orientation of amino acid residues Arg174, Arg178, and Arg184 in apo form of formate dehydrogenase from *C.boidinii* (PDB structure 2FSS.PDB). (B). Removal of electrostatic repulsion and the production of a new hydrogen bond in CboFDH due to the amino acid change Arg178Ser.

The data on the effect that an increase in the phosphate buffer concentration has on the inactivation rate constant of the wild–type CboFDH and an analysis of the quaternary structure allow us to explain the dramatic stabilization effect of CboFDH in a 0.1 М phosphate buffer due to the Arg178Ser mutation observed in [11]. This substitution increases enzyme stability more than three–fold [2]. The mechanism behind this stabilization is as follows. Two more Arg residues are located near the latter residue in positions 174 and 182 (Fig. 5А). Their positively charged guanidine groups are situated 4.05 Å and 4.78 Å from Arg178, respectively. These are relatively large distances, and the repulsive forces will not be too significant in the case of only one pair of positive residues; however, there are two pairs of such residues, so the effectiveness of the repulsion is improved dramatically. The Arg178Ser substitution diminishes the electrostatic repulsion between the positive charges, and a new hydrogen bond is formed between the Arg182 and Ser178 residues (Fig. 5B), which is the reason for the high stability of an enzyme with this substitution. Increasing the concentration of the phosphate buffer masks the positive charges with the negatively charged phosphate ions.

A comparison of bacterial, plant, yeast, and fungi FDH amino acid sequences indicates that arginine residues which correspond to the CboFDH Arg174 and 182 residues are conserved in all of the above–mentioned enzymes (see Fig. 1 in ref. [2]). Moreover, the Arg174 residue is part of the ** (G/A) ** a ** G ** ir ** G region, which is a “fingerprint” sequence fo coenzyme binding ** domain in dehydrogenases. An X–ray analysis for * Pseudomonas * sp.101 [3] and * Moraxella * sp.C2 FDH showed that the arginine residue from the signature region (Arg202 in bacterial enzymes) is involved in the binding of NAD^+^, interacting with its pyrophosphate group. It is obvious that substituting these two conserved Arg residues (especially Arg174) should disrupt the enzyme’s catalytic functions. In yeast and fungi, the Arg178 residue of FDH is completely conserved. However, bacteria and higher plant FDH have either Ala or Leu residues, respectively, in the position corresponding to Arg178 in the amino acid sequence of CboFDH. The reason that nature “chose” to put a disadvantageous arginine residue into this position in yeast and fungi FDH remains unclear, and further investigation is needed to provide an answer to this question.

## The Dependence of CboFDH Thermal Stability on Sodium Formate Concentration

**Fig. 6:**
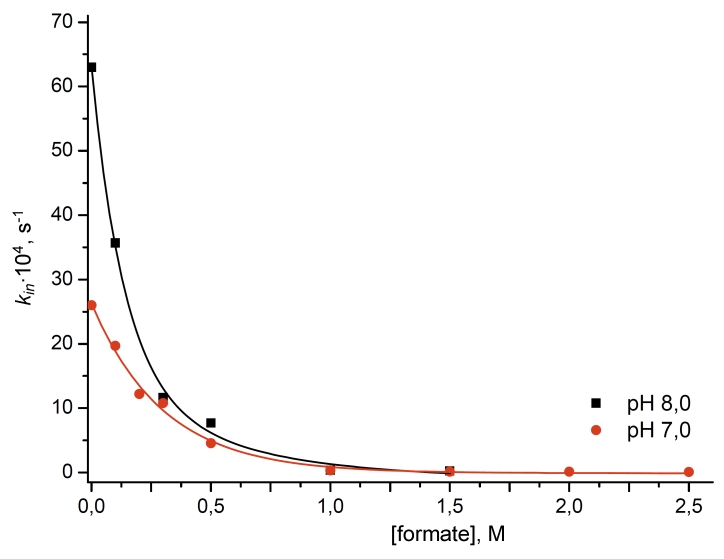
Influence of the sodium formate concentration on the inactivation rate constant of CboFDH in a 0.1M sodium phosphate buffer at pH 7.0 and 61°C and at pH 8.0 and 59°C.

Formate dehydrogenase is widely used in dehydrogenase catalyzed synthesis of chiral compounds as a coenzyme regenerating catalyst, and high concentrations (up to 2–3 M) of formate–ion, substrate of FDH, are used to achive high turnover of coenzyme. This is why we decided to examine CboFDH thermal inactivation kinetics upon varying sodium formate concentrations at two pH values: 7.0 and 8.0 (Fig. 6) since these are the values which are most often used for enzymatic synthesis processes involving dehydrogenases. As can be seen in Fig. 6, the dramatic stabilization of the enzyme is observed at high concentrations of sodium formate. This effect is especially notable at concentrations of sodium formate reaching 1.5 М. As in the case of the relation between the inactivation rate constant and the concentration of the phosphate ion, the stabilization effect is more pronounced at рН 8.0 than at р Н 7. 0 (Fig. 6).

**Fig. 7:**
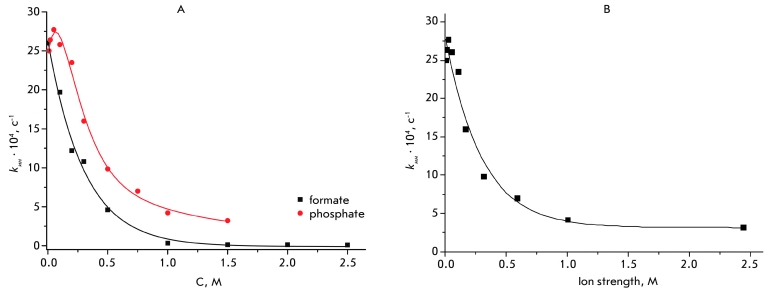
(A) Influence of the concentrations of the sodium phosphate buffer and sodium formate on the inactivation rate constant of CboFDH. pH 7.0 and 61°C. (B) Influence of the sodium phosphate buffer concentration presented as the ion strength value on the inactivation rate constant of CboFDH at pH 7.0 and 61°C.

A comparison of the relations between the inactivation rate constants and the phosphate and formate concentrations (Fig. 7A) obtained at рН 7.0 and 61°С shows that formate is better than phosphate at stabilizing CboFDH; moreover, achieving this stabilization requires much lower concentrations of substrate. However, a recalculation of the phosphate concentration for the according ionic strength indicates that the relation between the inactivation rate constant and ionic strength is identical, and this relation can be described as a simple exponent, just as in the case of the formate ion (Fig. 7B). This fact suggests a universal CboFDH stabilization effect under the influence of ionic strength. The more effective stabilization in the case of formate is most likely due to the fact that the formate ion is smaller and can thus penetrate deeper into the protein globule or bind specifically.

The data on CboFDH stabilization at high concentrations of formate can be used in practice for the enzymatic synthesis of chiral compounds. Moreover, CboFDH can be stored in buffer solutions with high salt concentrations at +4 °С without a significant loss of activity , as compared to the usual conditions, –20 °С in 40% glycerine. The high salt concentration also lowers the concentration of oxygen in the solution, which gives the enzyme additional protection from inactivation due to the oxidation of sulfhydryl groups of cystein residues. In our case, preparations of recombinant wild–type CboFDH were stored for 9 months with no loss of activity in a solution of ammonium sulfate (35% of saturated solution) and a 0.1 М phosphate buffer, рН 7.0 at a temperature of 4 °С .

In conclusion, we must note that the results of a direct comparison between the thermal stability of bacterial and yeast FDH, based on a study of inactivation kinetics, are in agreement with the thermal stability data determined for these enzymes by differential scanning calorimetry, which were also obtained in our laboratory [12] and used the same preparations of recombinant CboFDH as those used in this work.

Thus, in this work, the first systematic study of the thermal stability of recombinant * C. boidinii * formate dehydrogenase was performed. The resulting data allowed a direct comparison between this enzyme and the FDH from * Pseudomonas * sp.101 bacterium. The obtained data indicate that the inactivation mechanism for these enzymes is identical, but the effect of the surroundings on their stability differs.
